# Reviews

**Published:** 1882-08

**Authors:** 


### Reviews.

---

**Trance and Muscle Reading.** By GEORGE M. BEARD, A. M., M. D. New York.

The forty pages of this pamphlet relate to a subject which might well occupy many times the same space, and yet the principal points are well presented and considered in all fairness. The essay furnishes entertaining reading for any one interested in such studies.

---

**The Physician Himself, and What He should Add to the Strictly Scientific.** By D. W. CATHELL, M. D. Baltimore: Cushings & Bailey. Price, $1.25.

A series of excellent homilies, this is, which remind the practitioner of his shortcomings, and show him what he should be as a man and a citizen. The gentlemanly, upright physician is apt to see the straightforward path without any such directions, and one possessed of moral obliquity, would not be apt to follow it, even if it were pointed out. None the less is it well to have this done, and the writer has here succeeded in making prominent many a point in regard to tact and business management, which is of enormous importance in directing the course of what the world calls a "successful physician."

---

**The Incidental Effects of Drugs.** By DR. L. LEWIN. Translated by W. T. ALEXANDER, M. D. New York: William Wood & Co.

It is well that we are taught occasionally as to how much, or rather how little, can be expected from drugs already known. This is, perhaps, better than additions to our list, already too long. At any rate, the writer has here clearly presented the principles upon which many familiar drugs act, and in addition gives numerous suggestions which can be made use of clinically. An excellent book it is for practitioner as well as student.

**Marriage and Parentage.** By a "Physician and Sanitarian." New York: M. L. Holbrook & Co.

The drift of this series of articles is to show that men and women ought to have their senses about them, when thinking seriously of matrimony. A sound proposition, this, and one in which most persons—especially those already married—will, doubtless, concur. But when the writer goes so far as to advocate that young people should "consult a wise sanitarian before entering into the marriage relation," he oversteps the limit of public opinion, to say the least. This would, undoubtedly, be very advantageous to professional sanitarians, as they could then abjure sewer gas and similar nauseating subjects, and turn their attention to the more attractive arts of match-making.—Probably this suggestion of the writer will not be carried out just at present, but many others are made which are sensible and practical. In general it is an excellent little book for physicians or others contemplating marriage. To such we earnestly recommend it.

---

**Diseases of the Ear in Children.** By ANTON VON TROELTSCH, M. D. Translated by J. Orne Green, A. M., M. D. New York: William Wood & Co.

This originally formed the contribution of von Troeltsch to Gerhardt's Manual of Children's Diseases, and as now presented in a seperate volume, must prove acceptable to many who do not care to purchase that entire work. It comes from the pen of one of the highest authorities in otology in Germany, and is translated by another who occupies the same position in this country. This of itself, is sufficient recommendation. Of course, the book contains much that relates to diseases of the ear in adults, but it is written from a standpoint which enables the practitioner to judge more from signs than from symptoms, and in this it is especially of value. No one can read it without admiring the erudition of the author and profiting by his practical suggestions.

